# Risk factors for neuromuscular complications in lower limbs after lung transplantation

**DOI:** 10.3389/fneur.2022.1066104

**Published:** 2022-12-06

**Authors:** Soomi Cho, Jee Eun Lee, Byeong Joo Choi, Song Yee Kim, Moo Suk Park, Hyo-Hyun Kim, Jin Gu Lee, Hyo Chae Paik, Ha Young Shin, Seung Woo Kim

**Affiliations:** ^1^Department of Neurology, Yonsei University College of Medicine, Seoul, South Korea; ^2^Division of Pulmonology and Critical Care Medicine, Department of Internal Medicine, Severance Hospital, Yonsei University College of Medicine, Seoul, South Korea; ^3^Division of Cardiovascular Surgery, Severance Cardiovascular Hospital, Yonsei University College of Medicine, Yonsei University Health System, Seoul, South Korea; ^4^Department of Thoracic and Cardiovascular Surgery, Yonsei University College of Medicine, Seoul, South Korea

**Keywords:** lung transplant, extracorporeal membrane oxygenation, postoperative leg weakness, neuromuscular complications, risk factors

## Abstract

**Objective:**

This study aimed to analyze the prevalence and risk factors of neuromuscular complications after lung transplantation (LT), as well as the association between neuromuscular complications and extracorporeal membrane oxygenation (ECMO) support.

**Methods:**

We retrospectively included 201 patients who underwent LT between 2013 and 2020. Patients were classified into three groups based on the presence and the pattern of postoperative leg weakness: no weakness group, asymmetric weakness group, and symmetric weakness group. Comorbidities, duration of ECMO therapy, and postoperative complications were compared between the three groups.

**Results:**

Of the 201 recipients, 16 (8.0%) and 29 (14.4%) patients developed asymmetric and symmetric leg weakness, respectively. Foot drop was the main complaint in patients with asymmetric weakness. The presumed site of nerve injury in the asymmetric weakness group was the lumbosacral plexus in 8 (50%), peroneal nerve in 4 (25%), sciatic nerve in 2 (12.5%), and femoral nerve in 2 (12.5%) patients. In multivariate analysis, the use of preoperative ECMO was found to be independently associated with asymmetric weakness (OR, 3.590; 95% CI [1.227–10.502]). Symmetric leg weakness was associated with age at LT (1.062 [1.002–1.125]), diabetes mellitus (2.873 [1.037–7.965]), myositis (13.250 [2.179–80.584]), postoperative continuous renal replacement therapy (4.858 [1.538–15.350]), and duration of stay in the intensive care unit (1.052 [1.015–1.090]).

**Conclusion:**

More than 20% of patients developed leg weakness after LT. Early suspicion for peripheral neuropathy is required in patients after LT who used ECMO preoperatively, and who suffered from medical complications after LT.

## Introduction

Lung transplantation (LT) is a recognized treatment that improves the life expectancy and quality of life of patients with end-stage respiratory failure. Although survival after LT lags behind that of other solid organ transplants, the overall outcome has improved significantly (1). However, various unexpected complications pose significant threats to lung transplant recipients. Neurological complications following LT are common and cause substantial mortality (2–4). The incidence of neurological complications after LT is 79%, and the incidence of severe neurological complications is 38% (2). Most studies have focused on complications in the central nervous system, including stroke, seizure, and encephalopathy. Peripheral neuromuscular complications after LT have not been previously highlighted.

Extracorporeal membrane oxygenation (ECMO), a useful alternative to cardiopulmonary bypass, provides circulatory and respiratory support before, during, and after surgery (5). However, along with its widespread use, ECMO-related neurological complications are also being increasingly reported (6–8). Peripheral neuropathy is a neuromuscular complication related to ECMO that can profoundly affect the LT recipient's return to everyday life. However, few studies have focused on the occurrence of these complications in patients with LT.

Peripheral neuromuscular complications affecting lower extremity are common but possibly under recognized conditions in patients with LT. We aimed to elucidate the prevalence and risk factors for neuromuscular complications in a homogeneous group of patients who underwent LT. Additionally, we assessed whether neuromuscular complications were associated with the application of ECMO.

## Materials and methods

### Study populations

A retrospective analysis was performed on patients who underwent LT between March 2013 and June 2020 at Yonsei University Severance Hospital, Seoul, Republic of Korea. LT recipients were classified into three groups based on the presence and pattern of postoperative leg weakness attributed to neuromuscular diseases: (1) those with no clinical evidence of postoperative weakness (no weakness group), (2) those with asymmetric leg weakness (asymmetric weakness group), and (3) symmetric leg weakness (symmetric weakness group). Patients were defined as having asymmetric weakness if (1) the difference in muscle strength between the two legs graded by the Medical Research Council (MRC) scale was two or greater in any one of the hip, knee, and ankle joints, and (2) asymmetry is confirmed in electrophysiological evaluation. Those who developed bilateral leg weakness after LT and had a difference in MRC scale of ≤1 in every joint of legs were defined as having symmetric weakness. The cause of weakness and asymmetricity was confirmed by electrophysiological diagnostic tests. The following patients were excluded: (1) patients with pre-existing limb weakness; (2) presence of conditions other than neuromuscular disease that may cause weakness (e.g., brain lesion or spinal cord lesion); (3) patients whose muscle strength could not be accurately reported or assessed because of concomitant life-threatening conditions or low levels of consciousness, (4) those who had weakness but did not undergo electrodiagnostic studies, and (5) those whose clinical and electrophysiological features did not match. The study protocol was approved by the Institutional Review Board of Severance Hospital (IRB No. 4-2020-0853).

### Data collection and definition

Demographic and clinical data associated with LT and data related to ECMO support were collected by reviewing medical records. Duration of stay in the intensive care unit (ICU) and major postoperative complications during ICU stay were also collected. Major postoperative complications included postoperative bleeding that required re-exploration, prolonged ventilator support requiring tracheostomy, severe renal insufficiency requiring dialysis, and severe primary graft dysfunction requiring ECMO. The presence of postoperative cardiac arrhythmia, hyperbilirubinemia, and culture-proven infection—known risk factors for neurological injury in adults supported with ECMO—were also investigated (7).

### Neurological assessment

During the study period, LT recipients were routinely consulted to the Department of Neurology before undergoing LT, and if limb weakness was suspected after the surgery. A detailed neurological examination was conducted and recorded by a neurologist. Muscle strength was graded using the MRC scale in the muscle groups of the bilateral shoulder, elbow, wrist, hip, knee, and ankle joints. The presence and degree of limb weakness was determined by reviewing medical reports drafted by a neurologist. The dates of symptom development and the presence of sensory symptoms were also recorded. The functional status of the patient was assessed using the modified Rankin scale (mRS) at the time of the electrophysiological studies, with scores indicating the following: 0 (no symptoms), 1 (no significant disability, can carry out usual activities), 2 (slight disability, unable to carry out all activities), 3 (moderate disability, require help), 4 (moderately severe disability, unable to walk unassisted), 5 (severe disability, bedridden), and 6 (expired).

### Electrophysiological evaluation

Motor nerve conduction studies were routinely performed on the median, ulnar, peroneal, and tibial nerves to assess terminal latency, compound muscle action potentials (CMAP), and motor nerve conduction velocity (NCV). Sensory nerve action potentials (SNAPs) and sensory NCV were routinely recorded from the median, ulnar, superficial peroneal, and sural nerves. Motor nerve conduction studies on the femoral nerves or a sensory nerve conduction study on saphenous nerves were additionally conducted in patients with proximal leg weakness. Needle electromyography was performed to detect fibrillation potentials and positive sharp waves, as well as to assess motor unit action potentials. Needle electromyography was routinely conducted in vastus lateralis, tensor fasciae latae, tibialis anterior, tibialis posterior, and medial gastrocnemius muscles of the more affected limb. When abnormal spontaneous activities were observed, same muscle group in less affected limb was evaluate with needle electromyography. Asymmetry in electrophysiological evaluation was defined as (1) difference of CMAP or SNAP amplitude of >50% between more and less affected limb, and/or (2) needle electromyography showing abnormal spontaneous activity only in the more affected limb. The final diagnosis was made by neuromuscular specialists based on the clinical symptoms and results of electrophysiological and imaging studies.

### LT and ECMO technique

Surgical techniques for LT and protocol for perioperative management at Severance Hospital have previously been reported in detail (5, 9). Veno-arterial ECMO for intraoperative cardiopulmonary support during LT has been routinely performed in our institution since March 2013. In addition, those who were initially on preoperative veno-venous ECMO changed to veno-veno-arterial ECMO before or during LT (10). Postoperative ECMO was maintained in patients with severe graft dysfunction. The femoral vein and femoral artery were the preferred cannulation sites for veno-arterial ECMO, and the femoral vein and internal jugular vein were preferred for veno-venous ECMO. Femoral cut-down approaches were used for femoral cannulation in all cases of veno-arterial ECMO support. Central cannulation or dual venous cannulation was performed when the femoral vessels were insufficient. The type and duration of preoperative, intraoperative, and postoperative ECMO were recorded by reviewing the medical records.

### Statistical analysis

Data are shown as numbers (percentages) for categorical variables and as median (interquartile range) or mean (±standard deviation) for continuous variables. Categorical data were analyzed using Fisher's exact test or Pearson's χ2 test. Continuous variables were compared using the Kruskal–Wallis test followed by Dunn's *post-hoc* test for non-parametric data or analysis of variance (ANOVA) followed by Bonferroni's *post-hoc* test for normally distributed data. Multiple logistic regression analyses were performed to identify risk factors associated with postoperative leg weakness between the no weakness and asymmetric weakness groups, and between the no weakness and symmetric weakness groups. The results were reported as odds ratios and 95% confidence intervals (CIs). Variables with a two-tailed *P*-value < 0.05 in univariate analysis were selected as covariates in multivariate analysis. All statistical analyses were performed using the Statistical Package for the Social Sciences version 26.0 software (SPSS Inc., Chicago, IL, USA).

## Results

**Figure 1:**
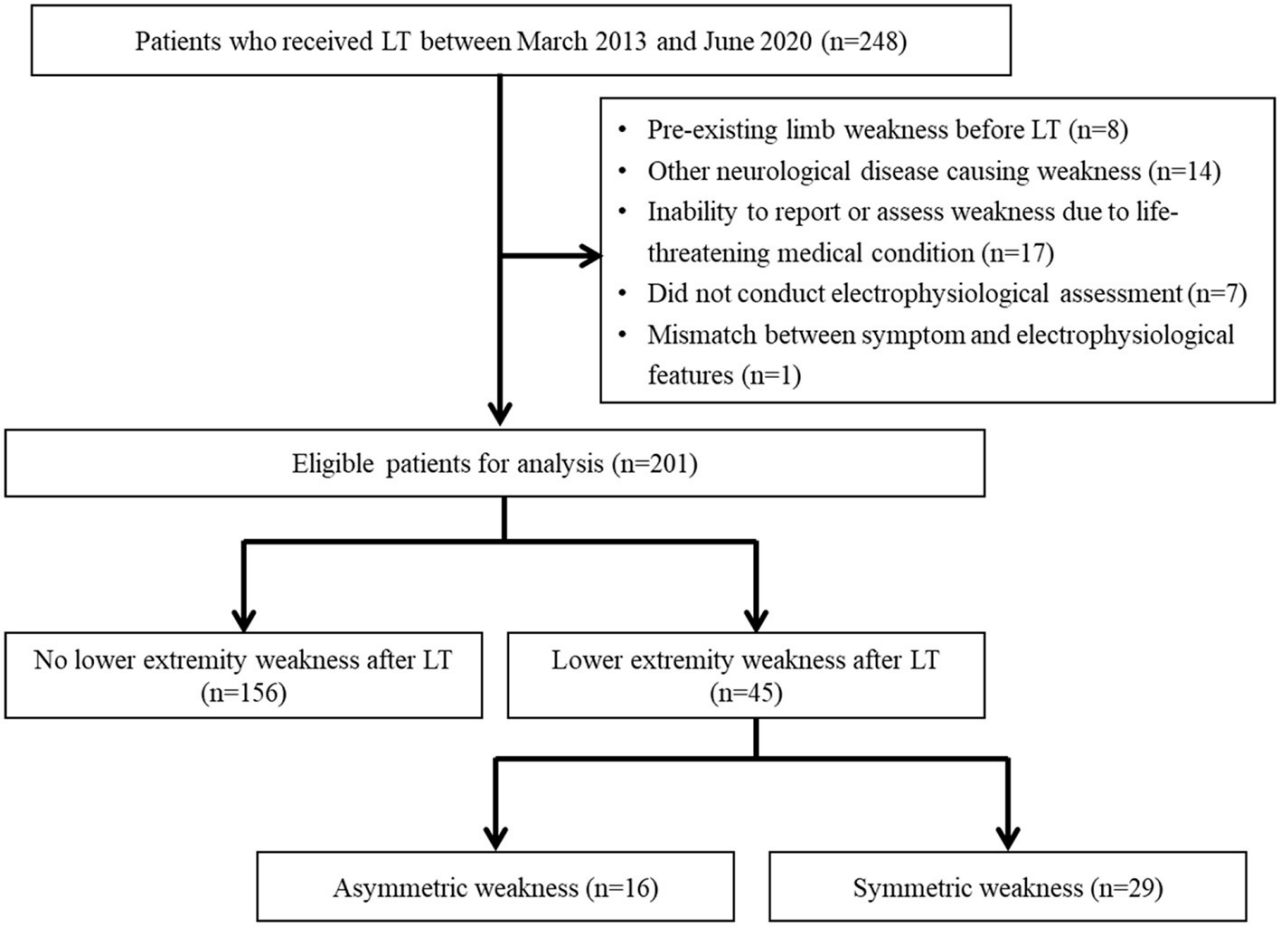
The study design and flow diagram of the patient selection process.

After excluding 47 patients who met the exclusion criteria, 201 LT recipients were included. Of the 201 patients, 156 (77.6%) had no postoperative leg weakness, 16 (8.0%) developed asymmetric leg weakness, and 29 (14.4%) developed symmetric leg weakness (Figure 1). The transplant procedures involved the bilateral lungs in 190 (94.5%) patients and the unilateral lung in 11 (5.5%) patients. Femoral arterial and venous cannulation was performed in 95.5 and 96.5% of patients, respectively.

### Comparison of demographic and clinical characteristics

The demographic and clinical characteristics of the groups are summarized in Table 1. There were no significant differences in recipient age or sex among the groups. The reason for LT was not significantly different between the groups, with idiopathic pulmonary fibrosis being the most common in all groups. In terms of comorbidities, the proportion of patients having history of myositis was significantly different among the groups (*p* = 0.021). In the *post-hoc* analysis, myositis tended to be more frequent in the symmetric weakness group (13.8%) than in the no weakness group (2.6%, *p* = 0.066). Preoperative ECMO was more frequently used in the asymmetric weakness group (50.0%, *p* = 0.04) or symmetric weakness group (44.8%, *p* = 0.015) than in the no weakness group (20.5%). Postoperative ECMO was more frequently used in the symmetric weakness group (62.1%) than in the no weakness group (37.8%, *p* = 0.045). The duration of intraoperative ECMO use was significantly longer in the asymmetric weakness group (median 6.2, Q1–Q3 [5.0–6.5] h) than in the no weakness group (5.0 [4.4–5.6] h, *p* = 0.020). The total duration of ECMO support was longer in the asymmetric weakness group (86.0 [6.8–460.4] h, *p* = 0.033) or symmetric weakness group (148.3 [36.8–325.7] h, *p*=0.001) than in the no weakness group (14.6 [4.8–83.5] h). There was no difference in the size of the arterial or venous cannula between the groups.

**Table 1:** Comparison of baseline clinical characteristics of lung transplantation recipients based on the presence and the pattern of postoperative leg weakness.

	**No weakness group** **(*n* = 156)**	**Asymmetric weakness group** **(*n* = 16)**	**Symmetric weakness group** **(*n* = 29)**	***P***
Age at lung transplant, years	55.5 (46.0–62.0)	58.5 (42.8–64.8)	60.0 (53.0–62.0)	0.130
Gender, male (%)	99 (63.5)	11 (68.8)	16 (55.2)	0.609
BMI, kg/m2	21.4 ± 3.5	19.9 ± 3.6	22.9 ± 4.0	0.025
**Reason for lung transplant**				0.474
IPF (%)	98 (62.8)	10 (62.5)	15 (55.2)	
CTD-ILD (%)	22 (14.1)	2 (12.5)	5 (17.2)	
COPD (%)	7 (4.5)	1 (6.3)	0 (0.0)	
Bronchiectasis (%)	9 (5.8)	1 (6.3)	2 (6.9)	
PHT (%)	2 (1.3)	0 (0.0)	1 (3.4)	
Others (%)	18 (11.5)	2 (12.5)	5 (17.2)	
**Comorbidities**				
Diabetes mellitus (%)	28 (18.0)	3 (18.8)	11 (37.9)	0.051
Chronic kidney disease (%)	4 (2.6)	0 (0.0)	3 (10.3)	0.160
Myositis (%)	4 (2.6)	1 (6.3)	4 (13.8)	0.021
Pre-operative ECMO use (%)	32 (20.5)	8 (50.0)	13 (44.8)	0.002 ^a, b^
Pre-operative ECMO duration, hrs	236.1 (169.9–412.0)	399.7 (147.0–590.2)	286.0 (175.9–489.4)	0.474
Intra-operative ECMO duration, hrs	5.0 (4.4–5.6)	6.2 (5.0–6.5)	5.6 (4.6–6.4)	0.005 ^a^
Post-operative ECMO use (%)	59 (37.8)	6 (37.5)	18 (62.1)	0.049 ^b^
Post-operative ECMO duration, hrs	39.8 (32.5–79.4)	72.1 (53.3–89.7)	61.0 (36.4–127.6)	0.109
Total ECMO duration, hours	14.6 (4.8–83.5)	86.0 (6.8–460.4)	148.3 (36.8–325.7)	< 0.001 ^a, b^
**Post-operative complications**				
Rethoracotomy	13 (8.3)	2 (12.5)	7 (24.1)	0.043
Tracheostomy	22 (14.1)	4 (25.0)	8 (27.6)	0.128
Pneumonia	43 (27.6)	7 (43.8)	15 (51.7)	0.023 ^b^
CRRT	12 (7.7)	0 (0.0)	8 (27.6)	0.005 ^b^
Hyperbilirubinemia	7 (4.5)	3 (18.8)	3 (10.3)	0.039
Atrial fibrillation/atrial flutter	34 (21.8)	5 (31.3)	11 (37.9)	0.151
Duration of total ICU stay, days	10.0 (6.0–18.3)	21.0 (10.0–25.8)	25.0 (15.0–32.0)	<0.001^a, b^

a Significant between the no weakness group and asymmetric weakness group.

b Significant between the no weakness group and symmetric weakness group.

BMI, body mass index; IPF, idiopathic pulmonary fibrosis; CTD-ILD, connective tissue disease-associated interstitial lung disease; COPD, chronic obstructive pulmonary disease; PHT, pulmonary hypertension; ECMO, extracorporeal membrane oxygenation; CRRT, continuous renal replacement therapy; ICU, intensive care unit.

### Comparison of postoperative complications

The incidence of reoperation for bleeding ligation, pneumonia, and continuous renal replacement therapy (CRRT) were significantly different across the groups (*p* = 0.043, *p* = 0.023, and *p* = 0.005, respectively), being the most frequent in the symmetric weakness group. In *post-hoc* analysis, the incidence of pneumonia and CRRT was more frequent in the symmetric leg weakness group (51.7 and 27.6%, respectively) than in the no weakness group (27.6 and 7.7%, *p* = 0.030 and *p* = 0.014, respectively). Although the incidence of hyperbilirubinemia was significantly different (*p* = 0.039), with it being the most frequent in the asymmetric weakness group, no significant difference was observed in the *post-hoc* analysis.

### Comparison of prognosis

We compared the duration of ICU stay and the duration of hospital stay after LT between the groups. The duration of ICU stay after LT was longer in the symmetric weakness group (14.0 [10.0–18.0] days) than in the asymmetric weakness group (8.5 [6.8–14.0] days) or no weakness group (7.0 [5.0–12.0] days, *p* < 0.001), but statistical significance was not observed in *post-hoc* analysis. The total duration of hospitalization after LT was significantly longer in the asymmetric weakness group (64.5 [38.5–99.3] days, *p* = 0.021) and symmetric weakness group (114.0 [60.0–145.0] days, *p* < 0.001) than in the no weakness group (36.0 [25.0–58.3] days). The in-hospital mortality rate was significantly higher in the symmetric weakness group (31.0%) than in the no weakness group (3.9%, *p* < 0.001). The mortality rate at 1 year after LT was also higher in the symmetric weakness group (41.4%) than in the no weakness group (18.6%, *p* = 0.02).

### Clinical features of patients who developed leg weakness after LT

The clinical features of patients with leg weakness are shown in Table 2. The median duration from LT to detection of leg weakness was 9.0 (3.3–16.0) days for the asymmetric leg weakness group, and 13.0 (9.0–21.5) days for the symmetric leg weakness group. Of the 16 patients with asymmetric leg weakness, 14 (87.5%) developed more severe leg weakness ipsilateral to the site of femoral ECMO cannulation. Foot drop was the chief complaint in 14 (87.5%) patients and MRC grade of ankle dorsiflexion was ≤3 in 13 (81.3%) patients. Weakness in knee extension and hip flexion was observed in 9 (56.3%) and 8 (50.0%) patients, respectively. MRC grade of knee extension was ≤3 in 4 patients. Symptoms of limb ischemia were observed in 1 patient (6.3%) in the asymmetric weakness group. All patients in both the symmetric and asymmetric weakness groups had an mRS score of 3 or worse at the time of the electrophysiological study. Eleven (38.0%) patients in the symmetric leg weakness group and none in the asymmetric weakness group were bedridden.

**Table 2:** Clinical features of patients who developed leg weakness after lung transplantation.

	**Asymmetric weakness group (*n* = 16)**	**Symmetric weakness group (*n* = 29)**	***P***
Duration from ICU admission to detection of weakness, days	23.1 ± 18.1	24.0 ± 11.1	0.830
Duration from lung transplant to detection of weakness, days	9.0 (3.3–16.0)	13.0 (9.0–21.5)	0.103
**Initial symptom**			
Weakness, *n* (%)	16 (100)	29 (100)	-
Numbness, *n* (%)	7 (43.8)	6 (20.7)	0.169
Tingling sensation, *n* (%)	3 (18.8)	11 (37.9)	0.313
Hypoesthesia, *n* (%)	16 (100)	24 (82.8)	0.144
Allodynia, *n* (%)	0 (0)	3 (10.3)	0.542
Edema, *n* (%)	5 (31.3)	6 (20.7)	0.483
**MRC grade at muscle group of maximal weakness**			0.233
Grade 1	6 (37.5)	4 (13.8)	
Grade 2	5 (31.3)	9 (31.0)	
Grade 3	4 (25.0)	9 (31.0)	
Grade 4	1 (6.3)	7 (24.1)	
**mRS at electrodiagnostic study**			0.017
3	9 (56.3)	9 (31.0)	
4	7 (43.8)	9 (31.0)	
5	0 (0)	11 (37.9)	

ICU, intensive care unit; MRC, Medical Research Council; mRS, modified Rankin Scale.

### Electrophysiological features of patients who developed asymmetric or symmetric leg weakness after LT

In the nerve conduction study, the amplitudes of peroneal CMAPs recorded from the extensor digitorum brevis muscle were abnormal in all patients with asymmetric and symmetric weakness (Table 3). The rate of reduced posterior tibial CMAP was significantly higher in the symmetric weakness group (96.6%) than in those with asymmetric weakness (68.8%, *p* = 0.017). In contrast, more patients in the asymmetric weakness group (68.8%) had decreased amplitudes of superficial peroneal SNAP compared to the symmetric weakness group (34.5%, *p* = 0.027). In the asymmetric leg weakness group, the presumed site of nerve injury was the lumbosacral plexus in eight (50.0%) patients, peroneal nerve in four (25.0%) patients, sciatic nerve in two (12.5%) patients, and femoral nerve in two (12.5%) patients. In electromyography, abnormal spontaneous activities were observed in tibialis anterior muscle in 14 (87.5%) patients, medial gastrocnemius muscle in 9 (56.3%), vastus lateralis in 9 (56.3%), and tensor fasciae latae in 6 (37.5%) patients. A conduction block at the fibular head was not observed in any of the patients.

**Table 3:** Electrodiagnostic features of patients who developed leg weakness after lung transplantation.

	**Asymmetric weakness group^a^ (*n* = 16)**	**Symmetric weakness group (*n* = 29)**	***P***
**NCS parameters, nerve (recording site)**			
**Peroneal (EDB) CMAP**			-
Normal	0 (0)	0 (0)	
Abnormal	16 (100)	29 (100)	
**Posterior tibial (AHB) CMAP**			0.017
Normal	5 (31.3)	1 (3.4)	
Abnormal	11 (68.8)	28 (96.6)	
**Femoral (RF) CMAP** ^b^			
Normal	0		
Abnormal	2 (100)		
**Sural SNAP**			0.912
Normal	8 (50.0)	15 (51.7)	
Abnormal	8 (50.0)	14 (48.3)	
**Superficial peroneal SNAP**			0.027
Normal	5 (31.3)	19 (65.5)	
Abnormal	11 (68.8)	10 (34.5)	
**Saphenous SNAP** ^c^			
Normal	4 (40.0)		
Abnormal	6 (60.0)		
**Probable site of lesion**			
Lumbosacral plexus, *n* (%)	8 (50.0)	-	
Femoral nerve, *n* (%)	2 (12.5)	-	
Sciatic nerve, *n* (%)	2 (12.5)	-	
Peroneal nerve, *n* (%)	4 (25.0)	-	

NCS, nerve conduction study; EDB, extensor digitorum brevis; AHB, abductor hallucis brevis; RF, rectus femoris; CMAP, compound muscle action potential; SNAP, sensory nerve action potential.

a Result of electrophysiological study in legs with more severe weakness.

b Conducted in two patients with femoral neuropathy.

c Conducted in two patients with femoral neuropathy and eight patients with lumbosacral plexopathy.

### Risk factors for asymmetric or symmetric leg weakness after LT

The risk factors associated with asymmetric weakness compared with the no weakness group were assessed. In univariate analysis, preoperative ECMO support, postoperative hyperbilirubinemia, and total duration of ICU stay were found to be associated with asymmetric weakness (Table 4). In multivariate analysis, the use of preoperative ECMO was found to be independently associated with asymmetric weakness (OR, 3.590; 95% CI [1.227–10.502]). The risk factors associated with symmetric weakness compared to no weakness in the univariate analysis included older age at LT, higher body mass index, presence of diabetes mellitus or myositis, preoperative ECMO use, rethoracostomy, postoperative pneumonia or CRRT, and longer duration of ICU stay (Table 5). In multivariate analysis, age at LT (1.062 [1.002–1.125]), diabetes mellitus (2.873 [1.037–7.965]), myositis (13.250 [2.179–80.584]), postoperative CRRT (4.858 [1.538–15.350]), and duration of ICU stay (1.052 [1.015–1.090]) were independently associated with symmetric leg weakness.

**Table 4:** Risk factors for asymmetric leg weakness after lung transplantation.

	**Univariate**		**Multivariate†**	
	**HR (95% CI)**	***P***	**HR (95% CI)**	***P***
Pre-operative ECMO use	3.875 (1.350–11.120)	0.012	3.590 (1.227–10.502)	0.020
Hyperbilirubinemia	4.912 (1.133–21.289)	0.033	4.151 (0.901–19.113)	0.068
Duration of total ICU stay, days	1.043 (1.008–1.080)	0.017	1.018 (0.973–1.065)	0.435

HR, hazard ratio; CI, confidence interval; ECMO, extracorporeal membrane oxygenation; ICU, intensive care unit.

**Table 5:** Risk factors for symmetric leg weakness after lung transplantation.

	**Univariate**		**Multivariate†**	
	**HR (95% CI)**	***P***	**HR (95% CI)**	***P***
Age at lung transplant, years	1.046 (1.003–1.092)	0.037	1.062 (1.002–1.125)	0.041
BMI, kg/m2	1.120 (1.003–1.251)	0.043	1.088 (0.958–1.237)	0.195
Diabetes mellitus	2.794 (1.189–6.564)	0.018	2.873 (1.037–7.965)	0.042
Myositis	6.080 (1.428–25.894)	0.015	13.250 (2.179–80.584)	0.005
Pre-operative ECMO use	3.148 (1.375–7.210)	0.007	1.653 (0.505–5.412)	0.406
Rethoracotomy for bleeding ligation	3.500 (1.259–9.732)	0.016	2.178 (0.662–7.161)	0.200
Pneumonia	2.816 (1.254–6.320)	0.012	1.433 (0.498–4.128)	0.505
CRRT	4.571 (1.673–12.488)	0.003	4.858 (1.538–15.350)	0.007
Duration of total ICU stay, days	1.063 (1.030–1.097)	<0.001	1.052 (1.015–1.090)	0.006

HR, hazard ratio; CI, confidence interval; BMI, body mass index; ECMO, extracorporeal membrane oxygenation; CRRT, continuous renal replacement therapy; ICU, intensive care unit.

## Discussion

We investigated the incidence and clinical characteristics of neuromuscular complications during the early postoperative period from LT. More than 20% of patients develop neuromuscular complications after LT, which causes leg weakness that is often severe enough to inhibit independent ambulation. The risk factors for asymmetric weakness and symmetric weakness were different: asymmetric weakness was mainly associated with preoperative use of ECMO, whereas symmetric weakness was associated with comorbidities and poor postoperative medical condition.

Although not much has been studied regarding neuromuscular complications after LT, the present results are consistent with those of previous reports. In the present study, the approximate incidence of mono-neuropathy or lumbosacral plexopathy was 8.0%, and that of critical illness polyneuromyopathy (CIPM) was 14.4% among LT recipients. Živković et al. showed that 21.2% of LT recipients developed neuromuscular complications, including monon-europathies (7%), plexopathies (1.5%), polyneuropathies (8%), and myopathies (5%) (11), which is in line with the present results. Gamez et al. reported that CIPM is the most prevalent complication after LT, with an incidence of 29.6% (12). Although the incidence was not as high, CIPM was the most frequent neuromuscular complication in the present study. In a study by Gregoric et al. (13) foot drop developed in 7.8% of patients who underwent veno-arterial ECMO support. The incidence of foot drop in this report is very similar to the incidence of asymmetric weakness (8.0%) in the present study. In addition, the duration of veno-arterial ECMO support was significantly longer among patients who developed foot drop than those who did not, and foot drop developed on the ipsilateral side of arterial cannulation in 75% of the patients. These findings are also in line with the present results: the duration of ECMO support was significantly longer in the asymmetric weakness group than in the no weakness group, and asymmetric weakness developed in the ipsilateral side of the cannulation in 87.5% of patients.

Preoperative use of ECMO was the main risk factor for developing asymmetric leg weakness after LT. The association between ECMO and asymmetric leg weakness could be further supported by the fact that most patients with asymmetric leg weakness developed weakness in the ECMO-applied leg. Otherwise, no definite space-occupying lesion relevant to the patient's symptoms was identified despite extensive evaluations. Although common peroneal neuropathy may result from immobilization, a conduction block in the peroneal nerve was not observed in all patients. Thus, it is less likely that other clinical factors, including nerve compression associated with immobilization, iliopsoas muscle hematoma, or coexisting degenerative lumbar spine disease, may cause asymmetric weakness. We assume that the main mechanism of asymmetric neuropathy is limb ischemia associated with ECMO support. Arterial femoral cannulation may cause ischemia of the ipsilateral limb by reducing perfusion below the cannulation site. The proposed mechanism of limb ischemia includes venous stasis, arterial damage by cannulation, compression of the distal arteries, and accompanying hemodynamic instability. Paralysis and paresthesia are the characteristic clinical manifestations of limb ischemia along with cold, pulseless extremities (14). This suggests that the peripheral nerves and muscles can be damaged in patients with limb ischemia. The association between limb ischemia and peripheral neuropathy could be supported by previous cases of peroneal and tibial neuropathy resulting from limb ischemia after ECMO therapy and delayed reperfusion injury (15, 16).

Although limb ischemia is thought to be one of the main mechanisms of peripheral neuropathy associated with ECMO therapy, the possibility of other etiologies should be considered. Risk factors for limb ischemia in patients receiving ECMO therapy include a large cannula, young age, female sex, and diabetes mellitus (17). However, these factors were not significantly associated with asymmetric leg weakness in the present study. Additionally, symptoms of limb ischemia or acute compartment syndrome were observed in only 6.3% of patients with asymmetric weakness. Thus, a search for other etiologies is required if the symptoms of leg ischemia are not evident. Gregoric et al. (13) postulated that 7.8% of the patients with femoral cannulation for veno-arterial ECMO developed foot drop in the absence of limb ischemia. Although prolonged veno-arterial ECMO support was suggested to be a risk factor for foot drop, the influence of limb ischemia was thought to be minimal as patients with symptoms of limb ischemia were excluded. The authors suggested that prolonged immobility or impairment in circulation due to microvascular thrombosis or microembolization may have caused foot drop. Jang et al. reported a case of femoral neuropathy after veno-arterial ECMO therapy and postulated that direct nerve compression from the ECMO cannula may have caused neuropathy (18). Despite these hypotheses, the exact cause of unilateral limb weakness after ECMO therapy in the absence of limb ischemia is still uncertain, and further studies are required to elucidate the mechanism of neuropathy.

Patients who developed symmetric leg weakness could be diagnosed with CIPM, as limb weakness developed under the state of critical illness and neuromuscular involvement was confirmed by electrophysiological study, with other possible causes excluded (19). The risk factors for CIPM in the present study were old age, diabetes mellitus, myositis, postoperative CRRT, and length of stay in the ICU. Old age, hyperglycemia, renal replacement therapy, and long duration of organ dysfunction are known risk factors for CIPM (19). The presence of myositis is an additional risk factor for CIPM that should be considered in patients undergoing LT. Interstitial lung disease (ILD) is frequently accompanied by idiopathic inflammatory myositis (IIM) (20), and LT could be required in patients with refractory IIM-associated ILD. Patients with IIM-associated ILD are known to have a higher chance of graft dysfunction and longer duration of stay in the ICU than those without IIM (21), which may have led to a higher chance of CIPM. However, myositis was still independently associated with CIPM after adjusting for the duration of ICU stay or other medical complications. Although little is known regarding the association between IIM and the development of CIPM, underlying IIM could have made muscles prone to metabolic and microvascular alterations during the perioperative phase of LT, frequently leading to CIPM.

A nerve conduction study revealed that peroneal CMAP was below the normal range for our electrophysiological laboratory in all patients who developed leg weakness. Although posterior tibial CMAP amplitude was reduced in most cases with symmetric weakness, it was less frequently abnormal in the asymmetric weakness group. This finding is consistent with the clinical manifestation of asymmetric weakness, in which the chief complaint in most patients was foot drop. Of the sensory nerves, the sural SNAP amplitude was reduced equally in the asymmetric and symmetric weakness groups, whereas superficial peroneal SNAP amplitude was less frequently reduced in the symmetric weakness group. Based on these findings, it could be recommended to screen the peroneal and sural nerves of the ipsilateral side to ECMO cannulation to detect neuromuscular complications in patients with LT. Moss et al. previously demonstrated that combining the results of a nerve conduction study on the peroneal and sural nerves yielded the highest accuracy in identifying CIPM (22). As these nerves were also frequently damaged in patients with asymmetric weakness, the same technique could be recommended to screen for both asymmetric and symmetric weakness in patients undergoing LT.

This study has limitations common to retrospective studies, including risk of bias, especially due to misclassification or selection, and failure to measure other possible risk factors. In the present study, patients with mild symptoms perceived only by patients but not recognized by physicians may have been classified into the no weakness group if they did not report discomfort after LT. Additionally, leg weakness after LT could be an underdiagnosed condition in the intensive care setting. Use of sedatives and restriction of mobility during ICU stay make it difficult to detect neurological changes and perform exact neurologic examination.

## Conclusion

The present study assessed the incidence of neuromuscular complications in a homogeneous group of patients who underwent LT. More than 20% of patients developed leg weakness after LT. Besides critical illness polyneuromyopathy, 8% of the patients developed asymmetric leg weakness, mostly in the absence of limb ischemia. Preoperative ECMO support and a longer duration of intraoperative ECMO support were associated with asymmetric leg weakness. Close monitoring to detect limb weakness in these patients is required, as peripheral neuromuscular complications are associated with increased duration of hospitalization and mortality.

## Data availability statement

The raw data supporting the conclusions of this article will be made available by the authors, without undue reservation.

## Ethics statement

The studies involving human participants were reviewed and approved by Institutional Review Board of Severance Hospital. Written informed consent for participation was not required for this study in accordance with the national legislation and the institutional requirements.

## Author contributions

SC provided the conception and design of the study, acquisition of data, statistical analysis, interpretation of data, and drafting the manuscript. JEL and BJC supplied the acquisition of data and statistical analysis. SYK, MSP, H-HK, JGL, and HCP revised the manuscript critically for important intellectual content. HYS contributed to study design and interpretation of data. SWK provided the conception and design of the study, performed study supervision, and gave final approval of the version to be submitted. All authors contributed to the article and approved the submitted version.

## Funding

This study was supported by a faculty research grant of Yonsei University College of Medicine (6-2020-0069).

## Conflict of interest

The authors declare that the research was conducted in the absence of any commercial or financial relationships that could be construed as a potential conflict of interest.

## Publisher's note

All claims expressed in this article are solely those of the authors and do not necessarily represent those of their affiliated organizations, or those of the publisher, the editors and the reviewers. Any product that may be evaluated in this article, or claim that may be made by its manufacturer, is not guaranteed or endorsed by the publisher.
